# Autologous Stem-Cell-Based Gene Therapy for Inherited Disorders: State of the Art and Perspectives

**DOI:** 10.3389/fped.2019.00443

**Published:** 2019-10-31

**Authors:** Frank J. T. Staal, Alessandro Aiuti, Marina Cavazzana

**Affiliations:** ^1^Department of Immunohematology and Blood Transfusion (IHB), Leiden University Medical Center, Leiden, Netherlands; ^2^Paediatric Immunohematology Unit, San Raffaele Telethon Institute for Gene Therapy, IRCCS, San Raffaele Scientific Institute, Milan, Italy; ^3^Vita Salute, San Raffaele University, Milan, Italy; ^4^Biotherapy Department, Necker Children's Hospital, Assistance Publique-Hôpitaux de Paris, Paris, France

**Keywords:** gene therapy, SCID, thalassemia, sickle cell disease, gene editing, lysosomal storage disorder, clinical trial, curative treatment

## Abstract

Gene therapy using patient's own stem cells is rapidly becoming an alternative to allogeneic stem cell transplantation, especially when suitably compatible donors cannot be found. The advent of efficient virus-based methods for delivering therapeutic genes has enabled the development of genetic medicines for inherited disorders of the immune system, hemoglobinopathies, and a number of devastating metabolic diseases. Here, we briefly review the state of the art in the field, including gene editing approaches. A growing number of pediatric diseases can be successfully cured by hematopoietic stem-cell-based gene therapy.

## Introduction

Gene therapy refers to the introduction of nucleic acids (DNA or RNA) into target cells for therapeutic purposes. The objective is to either add a new copy of the “healthy” gene (additive gene therapy) or correct the mutated gene (gene editing). In principle, autologous gene therapy using hematopoietic stem cells (HSCs) as target cells provides an attractive alternative to allogeneic stem cell transplantation, since the gene therapy procedure will not trigger adverse events like graft-vs.-host disease or other immune complications (1–3). Autologous HSC-based gene therapy encompasses and broadens the indications for allogeneic hematopoietic cell transplantation (allo-HSCT); in addition to blood-specific diseases [e.g., primary immunodeficiencies (PIDs), hemoglobinopathies, congenital forms of cytopenia, and stem cell defects], metabolic diseases can also benefit from the cross-correction mechanism once the missing or aberrant protein is over-produced by circulating or tissue-resident mature blood cells. In the latter case, gene therapy can potentially work even better than allo-HSCT because the above-physiological levels of therapeutic gene expression can provide an often ubiquitous protein to the affected non-hematopoietic cells and tissues (as for mucopolysaccharidosis and Fabry disease, for example). Autologous HSC gene therapy leverages (i) more than 30 years of experience in bone marrow manipulation, and (ii) basic scientific knowledge about autologous and allo-HSCT, including the ease of isolating HSCs and hematopoietic stem progenitor cells (HSPCs) using CD34^+^ selection, and the important knowledge that HSCs home easily to their niches after intravenous (re)infusion. A large number of clinical trials have now provided robust evidence of multilineage engraftment and safe, stable transgene expression. Nevertheless, it remains to be seen whether truly permanent disease correction and long-term safety (i.e., beyond the current 20 years of hindsight) have been achieved. Moreover, the gene therapy approach may be limited by the high costs of vector manufacturing and cell transduction, and/or complex regulatory requirements. Precision approaches (based on gene editing with an endonuclease) are now entering the clinical arena (4).

## Vectors

**Figure 1:**
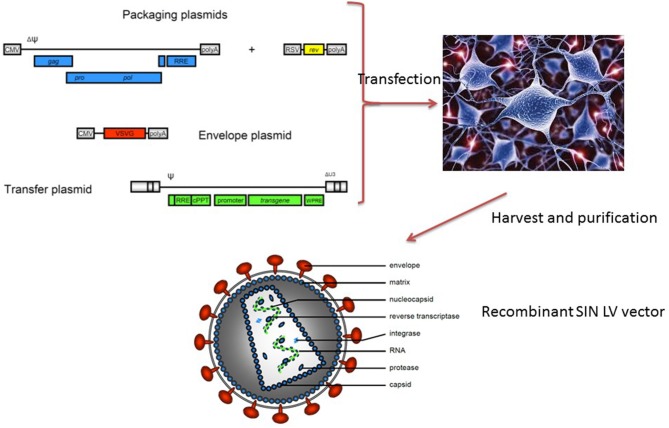
Current methods for generating lentiviral vectors. Four plasmids (a transfer vector containing the therapeutic gene and viral long terminal repeats, a REV-containing plasmid, a GAG-POL encoding plasmid, and an envelope-encoding plasmid, most often VSV-G) into a packaging line that subsequently secretes replication-deficient lentiviral particles. The latter are purified and then tested in several efficacy and safety assays before clinical use.

A large number of viral vectors have been used for gene therapy applications; retroviruses are usually used to transform HSCs because of their inherent ability to stably introduce viral genes into the host human genome (5). The retroviruses used in gene therapy have been rendered replication-deficient and have been stripped of all but the most important genes—some of which are provided via co-transfection with different plasmids into the so-called trans-complementing cell lines encoding the transgene construct and virus's structural components (gag, pol, and env) required for the production of infectious particles. Figure 1 illustrates the production of viral vectors for gene therapy applications. Stable packaging cell lines have been employed for gamma-retroviral vectors and, more recently, for HIV-derived lentiviral (LV) vectors (6). The first-generation vectors contained strong enhancers in their long terminal repeats (LTRs), so that transgene integration near cancer-associated genes resulted in unwanted gene transcription and insertional mutagenesis in all trials [for X-linked severe combined immune deficiency (SCID-X1), Wiskott–Aldrich syndrome (WAS), and chronic granulomatous disease (CGD)] except for those in adenosine deaminase SCID (ADA-SCID). These serious adverse events (discussed in more detail below) led to the development of a new generation of safer, self-inactivating (SIN) gamma-retroviral and LV vectors lacking potent enhancers in the LTRs. These vectors contain a transgene cassette whose expression is driven by internal ubiquitous promoters [e.g., Elongation Factor 1-alfa (EF) or phosphoglycerate kinase (PGK)] or tissue-specific promoters (7). These vectors are more difficult to produce but have two major advantages: the ability to transduce non-dividing cells, and less of a propensity to integrate near the start sites of actively transcribed genes. The LV vectors' pattern of integration into HSCs seems to be largely independent of the transgene encoded, and fewer proto-oncogenes are targeted (relative to gamma-retroviral vectors). Besides the integration pattern and the therapeutic gene, the main differences concern the choice of the promoter/enhancer elements that drive and regulate expression of the therapeutic gene. These choices depend on the pathophysiology of the disease and require significant preclinical work and toxicology studies in disease-specific animal models.

## SCID and Other PIDs

**Figure 2:**
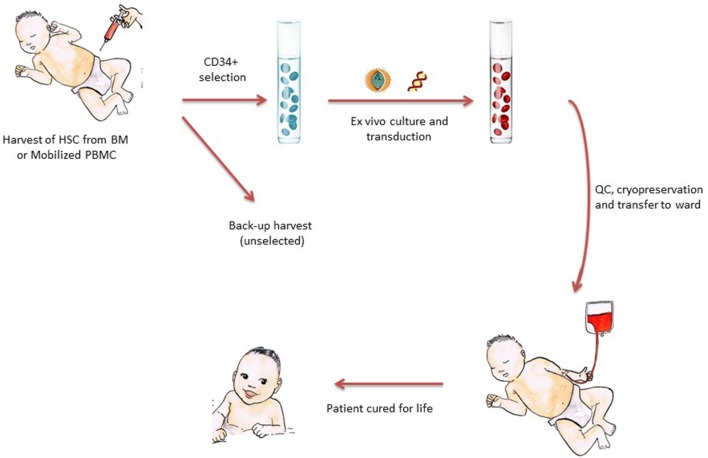
The principle of stem-cell-based gene therapy for pediatric diseases. CD34^+^ cells enriched for HSCs are harvested from the patients—either from bone marrow or (increasingly) from mobilized peripheral blood. The CD34^+^ cells are cultured in GMP laboratories with cytokines and viral vectors, harvested, and then subjected to a number of quality control steps prior to reinfusion into the patient. The cell product is often cryopreserved to allow time for quality control tests and the shipment of cells to clinical transplantation centers far from the production site. After reinfusion, HSCs find their niches, differentiate into mature blood cells, and thereby restore the clinical defect.

It is safe to say that the current clinical successes of gene therapy for inherited disorders and for cancer (e.g., using chimeric antigen receptor T lymphocytes) directly stem from the seminal work performed on gene therapy for SCID (8–11). Severe combined immunodeficiency is a rare, life-threatening disorder in which cells from the adaptive immune system do not develop properly. Patients with SCID are characterized by null or very low T-cell counts, due to an arrest in T-lymphocyte development (12). In most cases, the T-cell defect is combined with the absence or dysfunction of B-lymphocytes and/or innate cells (natural killer cells or neutrophils). Patients usually present with signs of SCID (severe and opportunistic infections, chronic diarrhea, and/or failure to thrive) in infancy. In the absence of treatment, most patients with SCID will die within the first year of life. As discussed in other chapters of this issue, allo-HSCT has been the curative treatment of choice for SCID for decades—especially when an HLA-compatible sibling donor is available (13, 14). The basic principle for most current pediatric gene therapy trials is shown in Figure 2. In short, CD34^+^ progenitor cells are harvested (from the patient's bone marrow or after mobilization in the circulation), transduced *ex vivo* with a viral vector, and then reinfused into the patient (as with any stem cell product).

The first clinical trial with gamma-retroviral vectors and gene-modified HSCs was designed by Bordignon et al. to correct ADA-SCID (9). Improvements in transduction protocols and the use of reduced-intensity conditioning enabled the successful treatment of ADA-SCID in Phase I/II trials and thus led to market authorization for this therapy (marketed as Strimvelis, discussed further below) for ADA-SCID by the European Medicines Agency (EMA) (15). All 27 treated patients are alive and show signs of therapy efficacy; 22 of them have shown robust immune reconstitution and have not required HSCT or enzyme replacement therapy post-treatment (16, 17). Similar results were obtained in other clinical trials (18, 19). In ongoing follow-up trials in the US and UK, a SIN LV vector is being used to treat ADA-SCID with promising results (16).

While gene therapy for ADA-SCID was being developed in Milan, gene therapy for X-linked SCID (caused by defects in the gamma chain of the interleukin 2 receptor) was developed initially in Paris (10) and then also in London (11). Despite major clinical benefits, genotoxic adverse effects occurred in Paris, London, and in all the other clinical trials of first-generation gamma retroviral vectors (with the exception of ADA-SCID trials). This led to the withdrawal of first-generation vectors and a return to the bench as part of an integrated international effort to understand these events and modify the vectors accordingly (20–22). Six of the first 20 SCID-X1 patients to be treated in these trials developed T-lymphocyte acute lymphoblastic leukemia (T-ALL) due to retroviral insertions near proto-oncogenes (mostly LMO2). The LMO2 gene is expressed in most hematopoietic cells but must be downregulated in the thymus during T-lymphocyte development; if not, leukemia can occur following a second hit (23, 24). In fact, the activation of these oncogenes serves as an initiating event in leukemia, which progresses after additional mutations occur in tumor suppressor genes or other oncogenes (22). It should be noted that five of the six patients with T-lymphocyte acute lymphoblastic leukemia (T-ALL) are in remission following successful chemotherapy, and one patient has died. It is still largely unclear why ADA-SCID gene therapy with gamma-retroviral vectors has been very successful so far, even though gene integrations near *LMO2* occurred in some patients (25, 26). Other possible explanatory factors are (i) the arrest of T-lymphocyte development caused by ADA deficiency, (ii) protection against leukemogenesis by non-transduced cells, and (iii) the fact that some *LMO2*-overexpressing human thymocytes show accelerated development rather than arrested development (27).

The development of second-generation SIN vectors has led to clinical success in the treatment of X-SCID (6, 28), ADA-SCID (18), WAS (29, 30), and X-CGD. An overview of pediatric diseases treated with gene-corrected CD34^+^ cells is given in Table 1. For the treatment of WAS (an immunodeficiency with immune dysregulation and thrombocytopenia), autologous HSCs were modified using an LV vector carrying the WAS cDNA driven by its own promoter. The interim results of three distinct clinical trials have shown evidence of multi-lineage engraftment, immunological improvement, a good safety profile, and protection against infections, autoimmune events, and bleeding (29, 30, 36).

**Table 1:** Ongoing clinical trials of gene therapy using autologous HSCs to treat inherited disorders in pediatric patients.

**Disease**	**Approach**	**Gene**	**Clinical study** **(clinicaltrials.gov)**
SCID-X1	LV	IL2RG	(6) NCT03311503 NCT03601286
ADA-SCID	LV	ADA	(16) NCT02999984 NCT02022696 NCT01852071 NCT03765632
Wiskott–Aldrich Syndrome	LV	WAS	(29, 30) NCT01347242 NCT01410825 NCT03837483
X-linked chronic granulomatous disease (CGD)	LV	Gp91phox	NCT01855685 NCT02757911 NCT02234934
Leucocyte adhesion deficiency (LAD)	LV	CD18	NCT03812263 NCT03825783
SCID due ARTEMIS defect	LV	DCLRE1C	NCT03538899
Transfusion dependent β-thalassemia	LV	HBB	(31, 32) NCT03207009 NCT02906202
Transfusion-dependent β-thalassemia	GE	HBB	NCT03728322
Sickle cell disease	LV	HBB	(33) NCT02140554 NCT03282656
Fanconi anemia	LV	FANCA	NCT03157804
Metachromatic leukodystrophy (MLD)	LV	ARSA	(34)
X-Adrenoleukodystrophy (ALD)	LV	ABCD1	(35)
Mucopolysaccharosidosis type I	LV	IDUA	NCT03488394

*LV, lentiviral vector; GE, gene editing*.

The same technologies are used for ongoing (Table 1) or planned trials for Artemis-SCID (37, 38), leukocyte adhesion deficiency due to CD18 mutations, and RAG1-SCID (39, 40)—all conditions for which preclinical studies have shown this procedure to be efficacious and safe (Table 1). For other types of SCID (e.g., IL7Rα-deficient SCID), it has become clear that regulated expression is essential; in fact, constitutive expression of the transgene prompts the development of leukemia due to the gene's signaling properties (and not because of insertional mutagenesis). Gene editing is needed (see below) for the treatment of these diseases. Although several other PIDs are candidates for gene therapy, many of them also require the regulation of gene expression. These include hyper IgM syndrome (where the *CD40L* gene is affected) (41) and X-linked a-gammaglobulinemia (XLA, where mutations in the *BTK* gene affect B-lymphocyte development at the pre B-lymphocyte stage) (42). In XLA, even LV vectors with B-lymphocyte-specific promoters lead to uncontrolled *BTK* expression in early progenitors and thus to the development of leukemia—despite the fact that the B-lymphocyte deficiency was restored in animal models of XLA (43). The hope is that gene editing approaches will preserve the normal gene regulation mechanisms and allow the correction of these diseases.

## Red Blood Cell Disorders

The two major disorders of erythrocyte development are β-thalassemia (resulting from low or null β-globin expression) and sickle cell disease (SCD, resulting from a specific point mutation in the *HBB* gene). Red blood cells from SCD patients display the characteristic sickle cell shape that is caused by polymerization of hemoglobin tetramers upon deoxygenation. These abnormally shaped cells can get trapped in blood capillaries, causing ischemia, multi-organ damage, and severe pain.

Given that these hemoglobinopathies are much more frequent than most PIDs and that few HLA-compatible donors are present in the international registry of bone marrow donors, there has been enormous impetus to develop gene therapy for these two red blood cell disorders (44, 45) (Table 1). Gene therapy for red blood cell disorders has benefited from better knowledge of their pathophysiology and advances in vector design in other gene therapy trials. Several different lentiviral technologies are therefore now being developed (46). Key success factors have been the incorporation of *HBB*'s locus control region (LCR) into LV vectors (47), and the deletion of the chromatin insulator in the LTR used in the first clinical trial for β-thalassemia. In fact, the presence of the chicken HS4 enhancer inserted in tandem in the first-generation LV vector revealed a cryptic splicing activity that triggered abnormal splicing of the proto-oncogene HMG-2A and thus led to benign clonal dominance in one of the treated patients (44). Using the new vectors, gene therapy for β-thalassemia has been continued in France and also initiated in Italy and the United States. In the seminal first report, a LV vector encoding an *HBB* gene with a β^T87Q^ mutation (known for its anti-sickling property) was placed under the transcriptional control of the β-globin promoter, a 3′ enhancer, and DNase-I hypersensitive sites 2, 3, and 4 from the β-globin LCR (48); this resulted in long-lasting β-globin expression. Similar approaches were used in two trials in the United States and one in Italy with a slightly different vector (referred to as GLOBE by the investigators) featuring an unmodified *HBB* gene and a different LCR (31).

Two recent interim trial reports reported the discontinuation or decrease in the requirement for long-term red blood cell transfusions in patients with β-thalassemia and the absence of adverse events related to gene therapy (31, 32). This is important because initial papers reported less successful outcomes because patients often remained transfusion dependent (45). The two studies differed with regard to the HSC administration route (intravenous vs. intrabone infusion), the conditioning regimen, and patients' age. A better clinical outcome was correlated with less severe (non-β^0^/β^0^) mutations and a higher level of *in vivo* engraftment by the gene-corrected cells. It is also possible that functional impairments in the bone marrow niche contributed to differences in the engraftment of gene-modified HSCs (46, 49). The EMA has now approved the first gene therapy for transfusion-dependent β-thalassemia in subjects aged 12 or less with non-severe β-globin mutations (i.e., non-β^0^/β^0^) and who lack an HLA-identical donor. It is clear that improved transduction efficiencies will be needed to provide sufficient *HBB* expression for the full correction of the most severe types of thalassemia. Gene editing approaches to the removal of fetal globin repressors (see below) are promising.

For patients with SCD, positive results have been obtained in trials in France (33) but other trials have showed more limited success (NCT02140554; NCT02186418). This suggests the need to optimize the source of the HSPCs, the dose of the transduced cells, the therapeutic level of transgene expression at the single progenitor level, and the type and pharmacokinetics of the conditioning regimen with a view to creating space for the transduced cells. The burdensome problem of harvesting low numbers of CD34^+^ cells from bone marrow under general anesthesia has been recently solved by the use of Plerixafor alone and then apheresis under very precise clinical circumstances in patients with SCD (50). Thanks to tightly controlling certain parameters, the most recent clinical results suggest that gene therapy for SCD has a promising future.

## Metabolic Diseases

Allo-HSCT is sometimes used to treat various metabolic diseases, and especially lysosomal storage diseases. The latter are caused by mutations in one of ~50 enzymes that break down large molecules (glycoproteins, lipids, glycogen, etc.) and pass the fragments on to other parts of the cell for recycling. Allo-HSCT has been used successfully to treat lysosomal storage diseases, and particularly mucopolysaccharidosis. The idea is that monocytes/macrophages can penetrate into many organs and thus supply the missing enzyme to various tissues, including bone, muscle, and brain. In this context, gene therapy with autologous cells has an additional advantage: depending on the vector's promoter, the therapeutic genes can be expressed at much higher levels than those normally present in HSCs and their offspring—thereby providing greater clinical benefit than allo-HSCT. This approach has produced spectacular results in patients with metachromatic leukodystrophy, with high-level production of the arylsulfatase enzyme in HSCs, mature blood cells, and central nervous system cells (34). This production prevented disease onset or halted disease progression, relative to untreated patients enrolled in a natural history study—especially when treatment was given before the onset of any clinical symptoms. In a clinical trial in X-linked adrenoleukodystrophy, the majority of patients had stable neurological function more than 2 years after gene therapy (35).

## Gene Editing vs. Gene Addition

**Figure 3:**
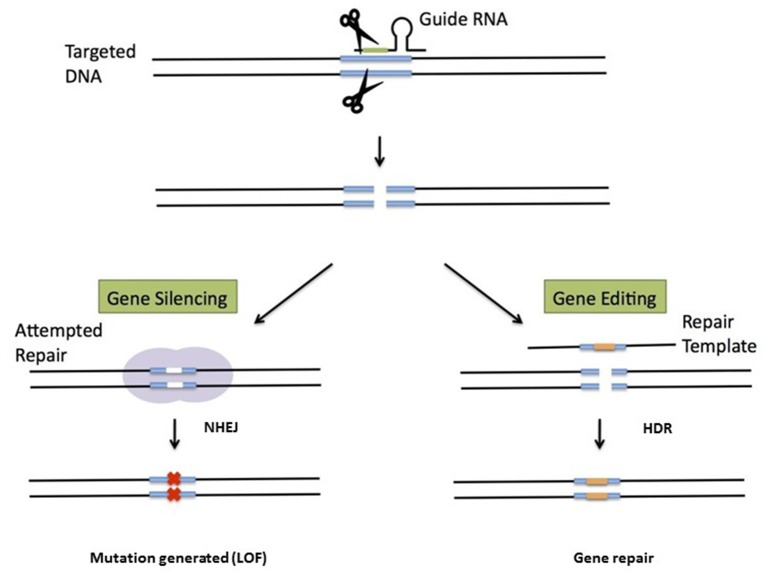
Genome editing for gene deletion or gene repair. A nuclease (often CAS9 with a guide RNA as part of the CRISPR system) generates double-strand DNA breaks. If a donor template is not provided, NHEJ will generate mutations that typically lead to a loss-of-function mutation. This strategy is used (for example) to remove repressors of fetal hemoglobin. The other approach requires a donor sequence for repair and relies on HDR. The donor template is often provided by an AAV.

Most of the gene therapy approaches discussed above rely on gene addition, i.e., the semi-random integration of one or more functional copies of a therapeutic gene into the host genome. As discussed above, gene addition is associated with a risk of insertional mutagenesis—even though not a single one of the 250 or so patients treated with LV vectors to date has experienced this type of adverse event. Hence, over recent years, investigators have started to test gene editing approaches that enable the *in situ* correction of mutant genes or the targeted integration of transgene cassettes into safe genomic harbors. Although the latter techniques do not regulate expression *per se*, expression patterns in target cells are predictable and consistent—thus reducing the risk of insertion near potentially dangerous sites. Site-specific genome modification is usually achieved by using enzymes that generate a double-strand break in the DNA near the mutation site (51). DNA repair enzymes than restore the break via either non-homologous end joining (NHEJ) or homology-directed repair (HDR). The former mechanism is quick but imprecise and thus leads to mutations via insertions, deletions, or rearrangements. In contrast, HDR repairs breaks precisely (as long as a template is available; see Figure 3) but depends on the cell cycle status. Several nuclease systems have been used to generate double-stranded breaks, including zinc finger nucleases (52), transcription activator-like effector nucleases (TALEN) (53), and Cas9 as part of the clustered regularly interspaced short palindromic repeat (CRISPR) system (54, 55). The versatile CRISPR system has become especially popular because it is good at recognizing target DNA sequences via a guide RNA. In some cases, simply generating a loss-of-function mutation has a beneficial effect, and only NHEJ is needed in such a case. In hemoglobinopathies, for instance, this strategy has been used to functionally disrupt the binding sites for the BCL11A protein that silences fetal HB expression (56). The negation of BLC11A's function can thus restore fetal HB expression and ameliorate the disease (57).

For most applications, however, a template DNA needs to be delivered to the cells along with the Cas9 protein. The transfection methods used for this purpose have improved tremendously for CD34^+^ cells, although 20–50% of cells still die during the procedure. The correction of long-term repopulating cells is still lower than those achieved with LV vectors (>90%). The template for HDR is then provided by an adeno-associated virus type 6 (AAV6) or an integration-deficient LV vector (58, 59). As most human disease mutations are spread out over the gene, specific gene repair is not a strategy that is easily applied to all affected patients. Therefore, most gene editing methods now simply insert a full cDNA of the therapeutic gene into the first exon of the locus (59), with the objective of maintaining normal regulation and normal expression levels. Preclinical work using nucleofection and template delivery with AAV6 is now being conducted for several PIDs: X-SCID (*IL2RG*) (60), hyper IgM syndrome (*CD40L*) (41), and XLA (*BTK*). For clinical application, a number of important issues with CRISPR gene editing must be tackled: (i) gene repair is not yet sufficiently effective, (ii) the occurrence and consequences of off-target side effects must be better understood, (iii) potential immune reactions to Cas9 or the gene product need to be overcome, and (iv) the process needs to be scaled up to clinically relevant numbers of CD34^+^ cells without triggering massive cell death. At present, HSCs must be cultured longer *ex vivo* for gene editing approaches than for gene addition approaches (2–4 vs. 1–2 days, respectively). Hence, for editing to be successful, better protocols for *ex vivo* expansion of HSCs and CD34^+^ cells need to be developed [for a review, see (61)]. Important, on-target side effects (microdeletions, translocations to different alleles, and incomplete insertions) caused by the CAS9 nuclease (62, 63) were recently identified as a major obstacle to clinical efficacy and regulatory approval. Despite these complex problems, it is clear that gene editing is an important pathway toward the development of treatments for a wide variety of target diseases. Clinical trials of gene editing approaches have already started (Table 1).

An exciting new development in the field of genomic editing is base editing, in which one base can be converted to another: a CG base pair becomes TA, or AT is converted to GC (64, 65). This nucleotide conversion happens without the need for the double-strand breaks generated by a fully active CRISPR–Cas9 system, and therefore does not invoke the corresponding DNA repair mechanisms. Base editing might be a very promising approach for correcting a disease caused by a single point mutation. However, safety, efficacy, side effects, and delivery methods must be better characterized and/or optimized before base editors can be used to treat patients.

## Future Perspectives

Gene therapy based on viral vectors is a relatively young science, and despite the great achievements, data on long-term efficacy (>10–20 years of observations) are still missing. Along with the promise of gene editing, non-viral delivery methods are likely to become more efficient and less toxic. For example, the methods currently used for nucleofection (e.g., for the delivery of CRISPR-CAs9 riboproteins) or the transfection of plasmids or RNA into CD34^+^ cells are still associated with significant toxicity in the targets. Various types of liposomes also are continuously being improved. Better non-viral delivery would enable a difficult step—namely, the very expensive GMP production of viral vectors—to be circumvented. However, it is still important to generate more efficient, scalable production platforms for high levels of viral vector production under GMP conditions. Furthermore, it is likely that better stem cell purification methods and *ex vivo* cell culture systems will be developed. For instance, clinically safe cell sorting would enable the purification of true HSCs, which account for no more than one in a 250 total CD34^+^ cells (66). The combination of immunoselection and transduction enhancers can reduce the amount of vector needed and potentially is safer and more cost-effective. For example, the addition of transduction-promoting polycations, such as polybrene and protamine sulfate (PS), is necessary in most protocols but have toxic effects that limit their use. Non-toxic alternatives, such as LentiBOOST™, an amphiphilic poloxamer, have the potential to increase efficiency and reduce vector requirements (67, 68).

In contrast to allo-HSCT, gene therapy is seen as a pharmaceutical drug, as Advanced Therapy Medicinal Products (ATMPs) entered via legislation into the pharma world. Although understandable, this has made development of gene therapy-based medicinal products much more complex, with long regulatory procedures and increased costs. Although pharmaceutical companies are needed for drug development, registration, pharmacovigilance, commercialization, and wider distribution, this process comes with a price tag. The price for Strimvelis was approved in Italy by the Italian Drug agency (AIFA), included in the special funds for innovative medicines with monitoring of the results for performance in the AIFA registry. Strimvelis is administered in Italy and available for all EU patients thanks to transborder EU legislation, reimbursed by the national health systems. The price for Zynteglo (autologous CD34^+^ cells transduced with the βA-T87Q-globin gene) and reimbursement is still under negotiation at present, but the company proposed publicly an installment plan over 5 years, with annual payments due only if the treatment continues to be effective.

The pricing for a curative medicine that is administered only once to a given patient is still subject to much debate. Fair pricing that societies can afford (for instance via health insurance reimbursement) must be balanced against the profits needed by biotech and pharma companies to survive (69). This may require new financial models that feature responsible entrepreneurship and access to treatments for patients. If gene therapy is to achieve its promise, these financial models may be as important as all the preclinical technical work being performed in laboratories worldwide.

## Author Contributions

All authors listed have made a substantial, direct and intellectual contribution to the work, and approved it for publication.

### Conflict of Interest

AA was the PI of clinical trials sponsored by Orchard Therapeutics. The remaining authors declare that the research was conducted in the absence of any commercial or financial relationships that could be construed as a potential conflict of interest. The handling editor AG declared a collaboration with one of the authors FS.

## References

[1] Dunbar CE High KA Joung JK Kohn DB Ozawa K Sadelain M. Gene therapy comes of age. Science. (2018) 359:eaan4672. 10.1126/science.aan4672 29326244

[2] Ginn SL Amaya AK Alexander IE Edelstein M Abedi MR. Gene therapy clinical trials worldwide to 2017: an update. J Gene Med. (2018) 20:e3015. 10.1002/jgm.3015 29575374

[3] Kohn DB. Gene therapy for blood diseases. Curr Opin Biotechnol. (2018) 60:39–45. 10.1016/j.copbio.2018.11.016 30599357

[4] Naldini L. Genetic engineering of hematopoiesis: current stage of clinical translation and future perspectives. EMBO Mol Med. (2019) 11:e9958. 10.15252/emmm.201809958 30670463 PMC6404113

[5] Staal FJ Pike-Overzet K Ng YY van Dongen JJ. Sola dosis facit venenum. Leukemia in gene therapy trials: a question of vectors, inserts and dosage? Leukemia. (2008) 22:1849–52. 10.1038/leu.2008.219 18769449

[6] Mamcarz E Zhou S Lockey T Abdelsamed H Cross SJ Kang G. Lentiviral gene therapy combined with low-dose busulfan in Infants with SCID-X1. N Engl J Med. (2019) 380:1525–34. 10.1056/NEJMoa1815408 30995372 PMC6636624

[7] Baum C von Kalle C Staal FJ Li Z Fehse B Schmidt M. Chance or necessity? Insertional mutagenesis in gene therapy and its consequences. Mol Ther. (2004) 9:5–13. 10.1016/j.ymthe.2003.10.013 14741772

[8] Aiuti A Cassani B Andolfi G Mirolo M Biasco L Recchia A. Multilineage hematopoietic reconstitution without clonal selection in ADA-SCID patients treated with stem cell gene therapy. J Clin Invest. (2007) 117:2233–40. 10.1172/JCI31666 17671653 PMC1934603

[9] Aiuti A Slavin S Aker M Ficara F Deola S Mortellaro A. Correction of ADA-SCID by stem cell gene therapy combined with nonmyeloablative conditioning. Science. (2002) 296:2410–3. 10.1126/science.1070104 12089448

[10] Cavazzana-Calvo M Hacein-Bey S de Saint Basile G Gross F Yvon E Nusbaum P. Gene therapy of human severe combined immunodeficiency (SCID)-X1 disease. Science. (2000) 288:669–72. 10.1126/science.288.5466.669 10784449

[11] Gaspar HB Parsley KL Howe S King D Gilmour KC Sinclair J. Gene therapy of X-linked severe combined immunodeficiency by use of a pseudotyped gammaretroviral vector. Lancet. (2004) 364:2181–7. 10.1016/S0140-6736(04)17590-9 15610804

[12] Wiekmeijer AS Pike-Overzet K H IJ Brugman MH Wolvers-Tettero IL Lankester AC. Identification of checkpoints in human T-cell development using severe combined immunodeficiency stem cells. J Allergy Clin Immunol. (2016) 137:517–26 e513. 10.1016/j.jaci.2015.08.022 26441229

[13] Bortin MM Rimm AA. Severe combined immunodeficiency disease. Characterization of the disease and results of transplantation. JAMA. (1977) 238:591–600. 10.1001/jama.1977.03280070031019 18618

[14] Buckley RH Schiff RI Schiff SE Markert ML Williams LW Harville TO. Human severe combined immunodeficiency: genetic, phenotypic, and functional diversity in one hundred eight infants. J Pediatr. (1997) 130:378–87. 10.1016/S0022-3476(97)70199-9 9063412

[15] Aiuti A Roncarolo MG Naldini L. Gene therapy for ADA-SCID, the first marketing approval of an *ex vivo* gene therapy in Europe: paving the road for the next generation of advanced therapy medicinal products. EMBO Mol Med. (2017) 9:737–40. 10.15252/emmm.201707573 28396566 PMC5452047

[16] Kohn DB Hershfield MS Puck JM Aiuti A Blincoe A Gaspar HB. Consensus approach for the management of severe combined immune deficiency caused by adenosine deaminase deficiency. J Allergy Clin Immunol. (2019) 143:852–63. 10.1016/j.jaci.2018.08.024 30194989 PMC6688493

[17] Cicalese MP Ferrua F Castagnaro L Pajno R Barzaghi F Giannelli S. Update on the safety and efficacy of retroviral gene therapy for immunodeficiency due to adenosine deaminase deficiency. Blood. (2016) 128:45–54. 10.1182/blood-2016-01-688226 27129325 PMC5325048

[18] Gaspar HB Cooray S Gilmour KC Parsley KL Zhang F Adams S. Hematopoietic stem cell gene therapy for adenosine deaminase–deficient severe combined immunodeficiency leads to long-term immunological recovery and metabolic correction. Sci Transl Med. (2011) 3:97ra80. 10.1126/scitranslmed.3002716 21865538

[19] Shaw KL Garabedian E Mishra S Barman P Davila A Carbonaro D. Clinical efficacy of gene-modified stem cells in adenosine deaminase-deficient immunodeficiency. J Clin Invest. (2017) 127:1689–99. 10.1172/JCI90367 28346229 PMC5409097

[20] Hacein-Bey-Abina S Garrigue A Wang GP Soulier J Lim A Morillon E. Insertional oncogenesis in 4 patients after retrovirus-mediated gene therapy of SCID-X1. J Clin Invest. (2008) 118:3132–42. 10.1172/JCI35700 18688285 PMC2496963

[21] Hacein-Bey-Abina S Von Kalle C Schmidt M McCormack MP Wulffraat N Leboulch P. LMO2-associated clonal T cell proliferation in two patients after gene therapy for SCID-X1. Science. (2003) 302:415–9. 10.1126/science.1088547 14564000

[22] Howe SJ Mansour MR Schwarzwaelder K Bartholomae C Hubank M Kempski H. Insertional mutagenesis combined with acquired somatic mutations causes leukemogenesis following gene therapy of SCID-X1 patients. J Clin Invest. (2008) 118:3143–50. 10.1172/JCI35798 18688286 PMC2496964

[23] Pike-Overzet K de Ridder D Weerkamp F Baert MR Verstegen MM Brugman MH. Gene therapy: is IL2RG oncogenic in T-cell development? Nature. (2006) 443:E5; discussion E6–7. 10.1038/nature05218 16988660

[24] Pike-Overzet K de Ridder D Weerkamp F Baert MR Verstegen MM Brugman MH Ectopic retroviral expression of LMO2, but not IL2Rgamma, blocks human T-cell development from CD34^+^ cells: implications for leukemogenesis in gene therapy. Leukemia. (2007) 21:754–63. 10.1038/sj.leu.2404563 17268520

[25] Aiuti A Cattaneo F Galimberti S Benninghoff U Cassani B Callegaro L. Gene therapy for immunodeficiency due to adenosine deaminase deficiency. N Engl J Med. (2009) 360:447–58. 10.1056/NEJMoa0805817 19179314

[26] Cassani B Montini E Maruggi G Ambrosi A Mirolo M Selleri S. Integration of retroviral vectors induces minor changes in the transcriptional activity of T cells from ADA-SCID patients treated with gene therapy. Blood. (2009) 114:3546–56. 10.1182/blood-2009-02-202085 19652199

[27] Wiekmeijer AS Pike-Overzet K Brugman MH van Eggermond MC Cordes M de Haas EF. Overexpression of LMO2 causes aberrant human T-cell development *in vivo* by three potentially distinct cellular mechanisms. Exp Hematol. (2016) 44:838–49.e9. 10.1016/j.exphem.2016.06.002 27302866

[28] De Ravin SS Wu X Moir S Anaya-O'Brien S Kwatemaa N Littel P. Lentiviral hematopoietic stem cell gene therapy for X-linked severe combined immunodeficiency. Sci Transl Med. (2016) 8:335ra357. 10.1126/scitranslmed.aad8856 27099176 PMC5557273

[29] Ferrua F Cicalese MP Galimberti S Giannelli S Dionisio F Barzaghi F. Lentiviral haemopoietic stem/progenitor cell gene therapy for treatment of Wiskott-Aldrich syndrome: interim results of a non-randomised, open-label, phase 1/2 clinical study. Lancet Haematol. (2019) 6:e239–53. 10.1016/S2352-3026(19)30021-3 30981783 PMC6494976

[30] Hacein-Bey Abina S Gaspar HB Blondeau J Caccavelli L Charrier S Buckland K. Outcomes following gene therapy in patients with severe Wiskott-Aldrich syndrome. JAMA. (2015) 313:1550–63. 10.1001/jama.2015.3253 25898053 PMC4942841

[31] Marktel S Scaramuzza S Cicalese MP Giglio F Galimberti S Lidonnici MR. Intrabone hematopoietic stem cell gene therapy for adult and pediatric patients affected by transfusion-dependent ss-thalassemia. Nat Med. (2019) 25:234–41. 10.1038/s41591-018-0301-6 30664781

[32] Thompson AA Walters MC Kwiatkowski J Rasko JEJ Ribeil JA Hongeng S. Gene therapy in patients with transfusion-dependent beta-thalassemia. N Engl J Med. (2018) 378:1479–93. 10.1056/NEJMoa1705342 29669226

[33] Ribeil JA Hacein-Bey-Abina S Payen E Magnani A Semeraro M Magrin E. Gene therapy in a patient with sickle cell disease. N Engl J Med. (2017) 376:848–55. 10.1056/NEJMoa1609677 28249145

[34] Sessa M Lorioli L Fumagalli F Acquati S Redaelli D Baldoli C. Lentiviral haemopoietic stem-cell gene therapy in early-onset metachromatic leukodystrophy: an *ad-hoc* analysis of a non-randomised, open-label, phase 1/2 trial. Lancet. (2016) 388:476–87. 10.1016/S0140-6736(16)30374-9 27289174

[35] Eichler F Duncan C Musolino PL Orchard PJ De Oliveira S Thrasher AJ. Hematopoietic stem-cell gene therapy for cerebral adrenoleukodystrophy. N Engl J Med. (2017) 377:1630–8. 10.1056/NEJMoa1700554 28976817 PMC5708849

[36] Scala S Basso-Ricci L Dionisio F Pellin D Giannelli S Salerio FA. Dynamics of genetically engineered hematopoietic stem and progenitor cells after autologous transplantation in humans. Nat Med. (2018) 24:1683–90. 10.1038/s41591-018-0195-3 30275570

[37] Benjelloun F Garrigue A Demerens-de Chappedelaine C Soulas-Sprauel P Malassis-Seris M Stockholm D. Stable and functional lymphoid reconstitution in artemis-deficient mice following lentiviral artemis gene transfer into hematopoietic stem cells. Mol Ther. (2008) 16:1490–9. 10.1038/mt.2008.118 18560421

[38] Lagresle-Peyrou C Benjelloun F Hue C Andre-Schmutz I Bonhomme D Forveille M. Restoration of human B-cell differentiation into NOD-SCID mice engrafted with gene-corrected CD34^+^ cells isolated from Artemis or RAG1-deficient patients. Mol Ther. (2008) 16:396–403. 10.1038/sj.mt.6300353 18223550

[39] Pike-Overzet K Baum C Bredius RG Cavazzana M Driessen GJ Fibbe WE. Successful RAG1-SCID gene therapy depends on the level of RAG1 expression. J Allergy Clin Immunol. (2014) 134:242–3. 10.1016/j.jaci.2014.04.033 25117803

[40] Pike-Overzet K Rodijk M Ng YY Baert MR Lagresle-Peyrou C Schambach A. Correction of murine Rag1 deficiency by self-inactivating lentiviral vector-mediated gene transfer. Leukemia. (2011) 25:1471–83. 10.1038/leu.2011.106 21617701

[41] Hubbard N Hagin D Sommer K Song Y Khan I Clough C. Targeted gene editing restores regulated CD40L function in X-linked hyper-IgM syndrome. Blood. (2016) 127:2513–22. 10.1182/blood-2015-11-683235 26903548

[42] Clough C Wang Y Khan IF Singh S Hung K Rawlings DJ 132. Targeting the BTK locus in primary human hematopoietic cells with TALENs and AAV donor template. Mol Ther. (2016) 24:S54 10.1016/S1525-0016(16)32941-0

[43] Ng YY Baert MR Pike-Overzet K Rodijk M Brugman MH Schambach A. Correction of B-cell development in Btk-deficient mice using lentiviral vectors with codon-optimized human BTK. Leukemia. (2010) 24:1617–30. 10.1038/leu.2010.140 20574453

[44] Drakopoulou E Papanikolaou E Georgomanoli M Anagnou NP. Towards more successful gene therapy clinical trials for beta-thalassemia. Curr Mol Med. (2013) 13:1314–30. 10.2174/15665240113139990064 23865429

[45] Nienhuis AW Persons DA. Development of gene therapy for thalassemia. Cold Spring Harb Perspect Med. (2012) 2:a011833. 10.1101/cshperspect.a011833 23125203 PMC3543108

[46] Lidonnici MR Paleari Y Tiboni F Mandelli G Rossi C Vezzoli M. Multiple integrated non-clinical studies predict the safety of lentivirus-mediated gene therapy for beta-thalassemia. Mol Ther Methods Clin Dev. (2018) 11:9–28. 10.1016/j.omtm.2018.09.001 30320151 PMC6178212

[47] Negre O Bartholomae C Beuzard Y Cavazzana M Christiansen L Courne C. Preclinical evaluation of efficacy and safety of an improved lentiviral vector for the treatment of beta-thalassemia and sickle cell disease. Curr Gene Ther. (2015) 15:64–81. 10.2174/1566523214666141127095336 25429463 PMC4440358

[48] Cavazzana-Calvo M Payen E Negre O Wang G Hehir K Fusil F. Transfusion independence and HMGA2 activation after gene therapy of human beta-thalassaemia. Nature. (2010) 467:318–22. 10.1038/nature09328 20844535 PMC3355472

[49] Crippa S Rossella V Aprile A Silvestri L Rivis S Scaramuzza S. Bone marrow stromal cells from beta-thalassemia patients have impaired hematopoietic supportive capacity. J Clin Invest. (2019) 129:1566–80. 10.1172/JCI123191 30830876 PMC6436882

[50] Lagresle-Peyrou C Lefrere F Magrin E Ribeil JA Romano O Weber L. Plerixafor enables safe, rapid, efficient mobilization of hematopoietic stem cells in sickle cell disease patients after exchange transfusion. Haematologica. (2018) 103:778–86. 10.3324/haematol.2017.184788 29472357 PMC5927997

[51] Jasin M. Genetic manipulation of genomes with rare-cutting endonucleases. Trends Genet. (1996) 12:224–8. 10.1016/0168-9525(96)10019-6 8928227

[52] Lombardo A Genovese P Beausejour CM Colleoni S Lee YL Kim KA. Gene editing in human stem cells using zinc finger nucleases and integrase-defective lentiviral vector delivery. Nat Biotechnol. (2007) 25:1298–306. 10.1038/nbt1353 17965707

[53] Cermak T Doyle EL Christian M Wang L Zhang Y Schmidt C. Efficient design and assembly of custom TALEN and other TAL effector-based constructs for DNA targeting. Nucleic Acids Res. (2011) 39:e82. 10.1093/nar/gkr739 21493687 PMC3130291

[54] Lin S Staahl BT Alla RK Doudna JA. Enhanced homology-directed human genome engineering by controlled timing of CRISPR/Cas9 delivery. Elife. (2014) 3:e04766. 10.7554/eLife.04766 25497837 PMC4383097

[55] Hendel A Bak RO Clark JT Kennedy AB Ryan DE Roy S. Chemically modified guide RNAs enhance CRISPR-Cas genome editing in human primary cells. Nat Biotechnol. (2015) 33:985–9. 10.1038/nbt.3290 26121415 PMC4729442

[56] Canver MC Smith EC Sher F Pinello L Sanjana NE Shalem O. BCL11A enhancer dissection by Cas9-mediated *in situ* saturating mutagenesis. Nature. (2015) 527:192–7. 10.1038/nature15521 26375006 PMC4644101

[57] Chang KH Smith SE Sullivan T Chen K Zhou Q West JA. Long-term engraftment and fetal globin induction upon BCL11A gene editing in bone-marrow-derived CD34(+) hematopoietic stem and progenitor cells. Mol Ther Methods Clin Dev. (2017) 4:137–48. 10.1016/j.omtm.2016.12.009 28344999 PMC5363298

[58] Maruyama T Dougan SK Truttmann MC Bilate AM Ingram JR Ploegh HL. Increasing the efficiency of precise genome editing with CRISPR-Cas9 by inhibition of nonhomologous end joining. Nat Biotechnol. (2015) 33:538–42. 10.1038/nbt.3190 25798939 PMC4618510

[59] Martin RM Ikeda K Cromer MK Uchida N Nishimura T Romano R. Highly efficient and marker-free genome editing of human pluripotent stem cells by CRISPR-Cas9 RNP and AAV6 donor-mediated homologous recombination. Cell Stem Cell. (2019) 24:821–8 e825. 10.1016/j.stem.2019.04.001 31051134

[60] Pavel-Dinu M Wiebking V Dejene BT Srifa W Mantri S Nicolas CE Gene correction for SCID-X1 in long-term hematopoietic stem cells. Nat Commun. (2019) 10:1634 10.1038/s41467-019-09614-y 30967552 PMC6456568

[61] Tajer P Pike-Overzet K Arias S Havenga M Staal FJT. *Ex vivo* expansion of hematopoietic stem cells for therapeutic purposes: lessons from development and the niche. Cells. (2019) 8:E169. 10.3390/cells8020169 30781676 PMC6407064

[62] Abou-El-Enein M Cathomen T Ivics Z June CH Renner M Schneider CK. Human genome editing in the clinic: new challenges in regulatory benefit-risk assessment. Cell Stem Cell. (2017) 21:427–30. 10.1016/j.stem.2017.09.007 28985524

[63] Cornu TI Mussolino C Cathomen T. Refining strategies to translate genome editing to the clinic. Nat Med. (2017) 23:415–23. 10.1038/nm.4313 28388605

[64] Abbasi J. DNA base editing could reverse most disease-causing point mutations. JAMA. (2017) 318:2173. 10.1001/jama.2017.19063 29234789

[65] Gaudelli NM Komor AC Rees HA Packer MS Badran AH Bryson DI. Programmable base editing of A^*^T to G^*^C in genomic DNA without DNA cleavage. Nature. (2017) 551:464–71. 10.1038/nature24644 29160308 PMC5726555

[66] Notta F Doulatov S Laurenti E Poeppl A Jurisica I Dick JE. Isolation of single human hematopoietic stem cells capable of long-term multilineage engraftment. Science. (2011) 333:218–21. 10.1126/science.1201219 21737740

[67] Delville M Soheili T Bellier F Durand A Denis A Lagresle-Peyrou C. A nontoxic transduction enhancer enables highly efficient lentiviral transduction of primary murine T cells and hematopoietic stem cells. Mol Ther Methods Clin Dev. (2018) 10:341–7. 10.1016/j.omtm.2018.08.002 30191160 PMC6125771

[68] Schott JW León-Rico D Ferreira CB Buckland KF Santilli G Armant MA. Enhancing lentiviral and alpharetroviral transduction of human hematopoietic stem cells for clinical application. Mol Ther Methods Clin Dev. (2019) 14:134–47. 10.1016/j.omtm.2019.05.015 31338385 PMC6629974

[69] Salzman R Cook F Hunt T Malech HL Reilly P Foss-Campbell B. Addressing the value of gene therapy and enhancing patient access to transformative treatments. Mol Ther. (2018) 26:2717–26. 10.1016/j.ymthe.2018.10.017 30414722 PMC6277509

